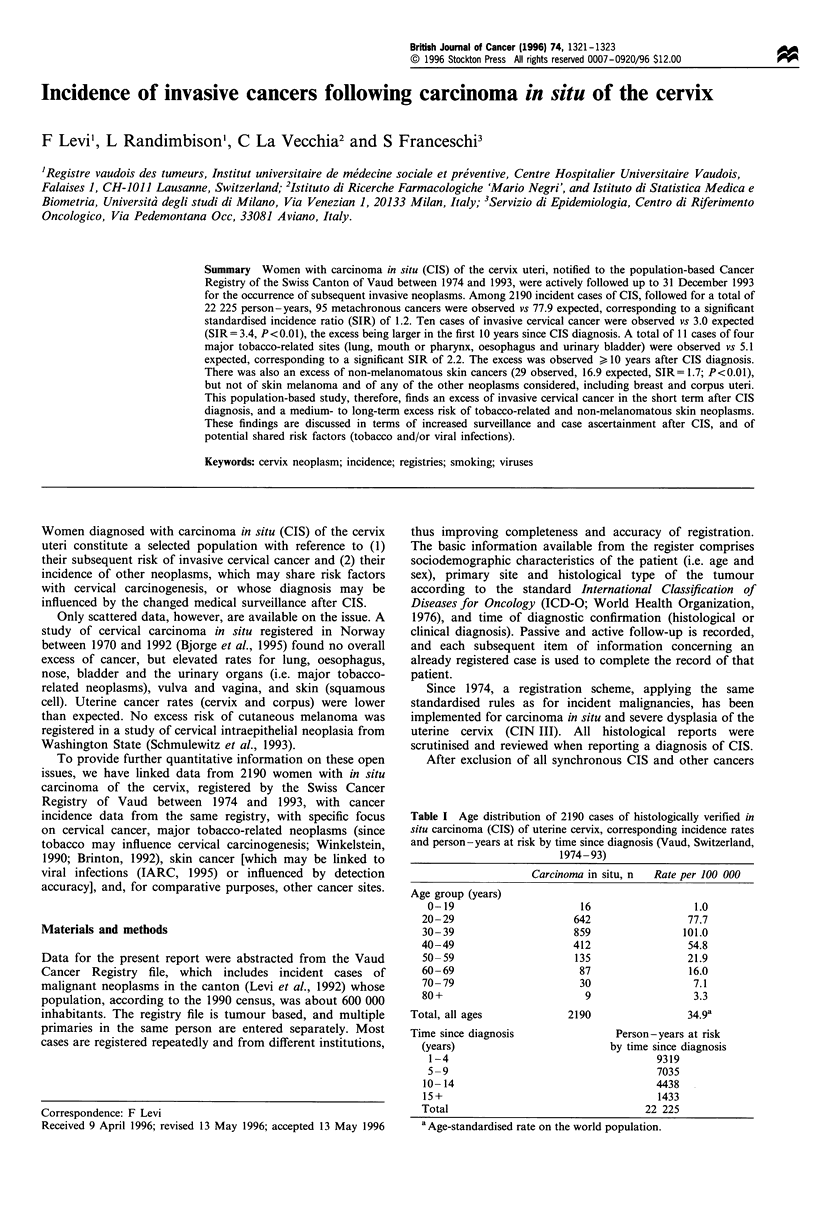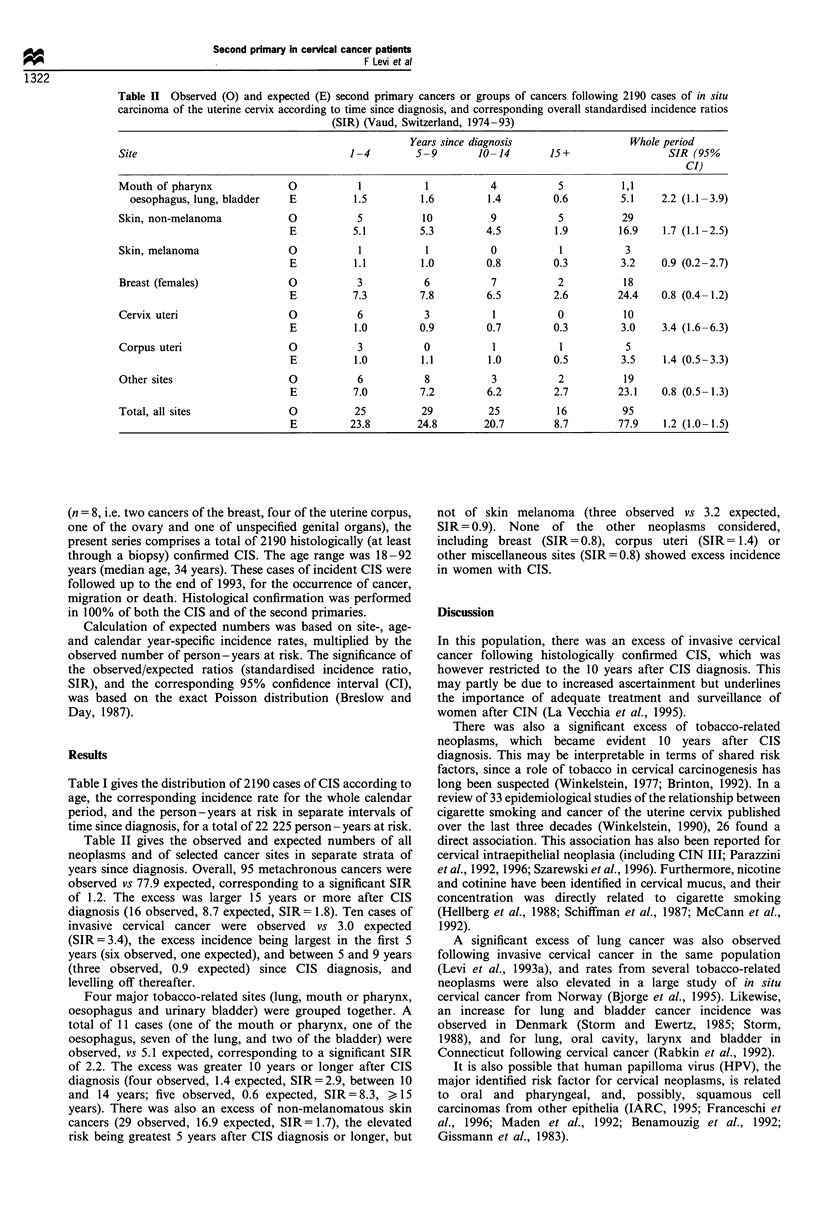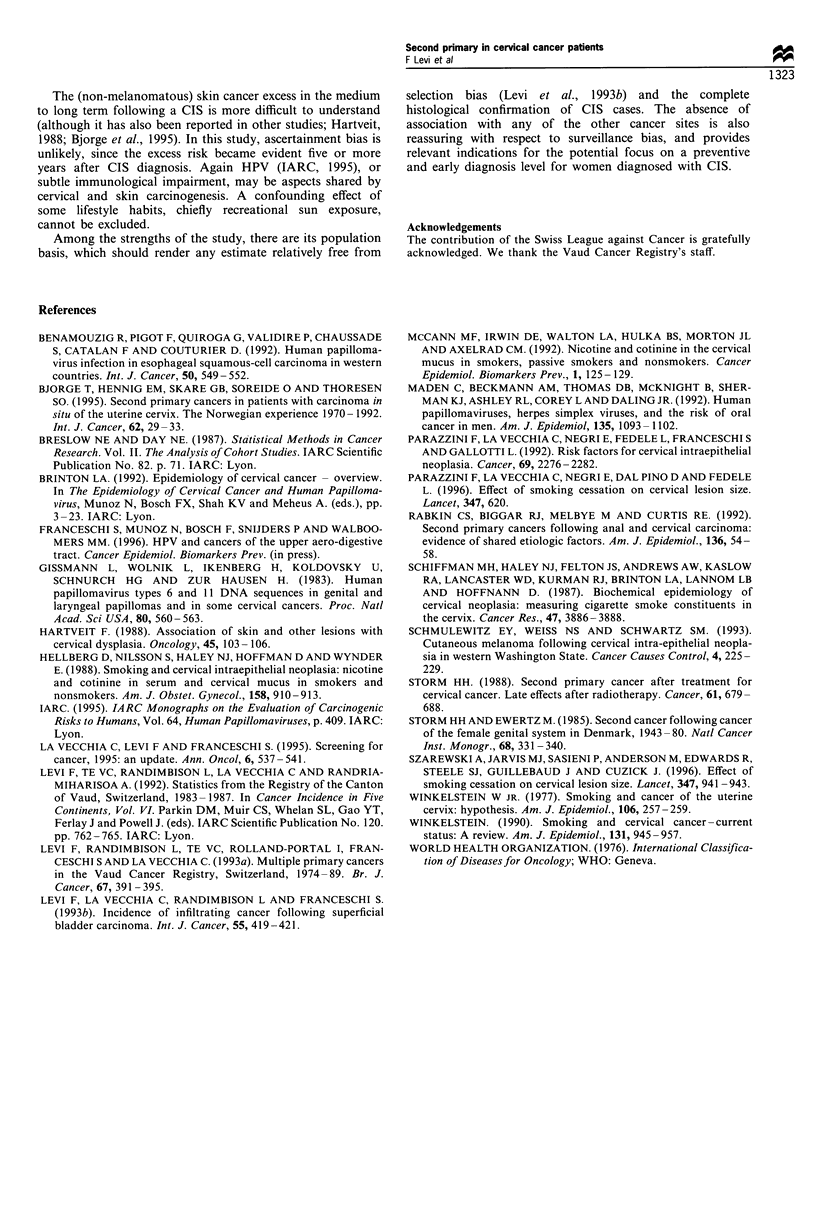# Incidence of invasive cancers following carcinoma in situ of the cervix.

**DOI:** 10.1038/bjc.1996.538

**Published:** 1996-10

**Authors:** F. Levi, L. Randimbison, C. La Vecchia, S. Franceschi

**Affiliations:** Institut universitaire de médecine sociale et préventive, Centre Hospitalier Universitaire Vaudois, Lausanne, Switzerland.

## Abstract

Women with carcinoma in situ (CIS) of the cervix uteri, notified to the population-based Cancer Registry of the Swiss Canton of Vaud between 1974 and 1993, were actively followed up to 31 December 1993 for the occurrence of subsequent invasive neoplasms. Among 2190 incident cases of CIS, followed for a total of 22,225 person-years, 95 metachronous cancers were observed vs 77.9 expected, corresponding to a significant standardised incidence ratio (SIR) of 1.2. Ten cases of invasive cervical cancer were observed vs 3.0 expected (SIR = 3.4, P < 0.01), the excess being larger in the first 10 years since CIS diagnosis. A total of 11 cases of four major tobacco-related sites (lung, mouth or pharynx, oesophagus and urinary bladder) were observed vs 5.1 expected, corresponding to a significant SIR of 2.2. The excess was observed > or = 10 years after CIS diagnosis. There was also an excess of non-melanomatous skin cancers (29 observed, 16.9 expected, SIR = 1.7; P < 0.01), but not of skin melanoma and of any of the other neoplasms considered, including breast and corpus uteri. This population-based study, therefore, finds an excess of invasive cervical cancer in the short term after CIS diagnosis, and a medium- to long-term excess risk of tobacco-related and non-melanomatous skin neoplasms. These findings are discussed in terms of increased surveillance and case ascertainment after CIS, and of potential shared risk factors (tobacco and/or viral infections).


British Journal of Cancer (1996) 74, 1321-1323
© 1996 Stockton Press All rights reserved 0007-0920/96 $12.00

### Incidence of invasive cancers following carcinoma *in situ* of the cervix

F Levi[1], L Randimbison[1], C La Vecchia[2] and S Franceschi[3]

*[1]Registre vaudois des tumeurs, Institut universitaire de médecine sociale et préventive, Centre Hospitalier Universitaire Vaudois, Falaises 1, CH-1011 Lausanne, Switzerland; [2]Istituto di Ricerche Farmacologiche 'Mario Negri', and Istituto di Statistica Medica e Biometria, Università degli studi di Milano, Via Venezian 1, 20133 Milan, Italy; [3]Servizio di Epidemiologia, Centro di Riferimento Oncologico, Via Pedemontana Occ, 33081 Aviano, Italy.*

**Summary** Women with carcinoma *in situ* (CIS) of the cervix uteri, notified to the population-based Cancer Registry of the Swiss Canton of Vaud between 1974 and 1993, were actively followed up to 31 December 1993 for the occurrence of subsequent invasive neoplasms. Among 2190 incident cases of CIS, followed for a total of 22 225 person-years, 95 metachronous cancers were observed *vs* 77.9 expected, corresponding to a significant standardised incidence ratio (SIR) of 1.2. Ten cases of invasive cervical cancer were observed *vs* 3.0 expected (SIR = 3.4, $P<0.01$), the excess being larger in the first 10 years since CIS diagnosis. A total of 11 cases of four major tobacco-related sites (lung, mouth or pharynx, oesophagus and urinary bladder) were observed *vs* 5.1 expected, corresponding to a significant SIR of 2.2. The excess was observed $\geqslant 10$ years after CIS diagnosis. There was also an excess of non-melanomatous skin cancers (29 observed, 16.9 expected, SIR = 1.7; $P<0.01$), but not of skin melanoma and of any of the other neoplasms considered, including breast and corpus uteri. This population-based study, therefore, finds an excess of invasive cervical cancer in the short term after CIS diagnosis, and a medium- to long-term excess risk of tobacco-related and non-melanomatous skin neoplasms. These findings are discussed in terms of increased surveillance and case ascertainment after CIS, and of potential shared risk factors (tobacco and/or viral infections).

**Keywords:** cervix neoplasm; incidence; registries; smoking; viruses

Women diagnosed with carcinoma *in situ* (CIS) of the cervix uteri constitute a selected population with reference to (1) their subsequent risk of invasive cervical cancer and (2) their incidence of other neoplasms, which may share risk factors with cervical carcinogenesis, or whose diagnosis may be influenced by the changed medical surveillance after CIS.

Only scattered data, however, are available on the issue. A study of cervical carcinoma *in situ* registered in Norway between 1970 and 1992 (Bjorge *et al*., 1995) found no overall excess of cancer, but elevated rates for lung, oesophagus, nose, bladder and the urinary organs (i.e. major tobacco-related neoplasms), vulva and vagina, and skin (squamous cell). Uterine cancer rates (cervix and corpus) were lower than expected. No excess risk of cutaneous melanoma was registered in a study of cervical intraepithelial neoplasia from Washington State (Schmulewitz *et al*., 1993).

To provide further quantitative information on these open issues, we have linked data from 2190 women with *in situ* carcinoma of the cervix, registered by the Swiss Cancer Registry of Vaud between 1974 and 1993, with cancer incidence data from the same registry, with specific focus on cervical cancer, major tobacco-related neoplasms (since tobacco may influence cervical carcinogenesis; Winkelstein, 1990; Brinton, 1992), skin cancer [which may be linked to viral infections (IARC, 1995) or influenced by detection accuracy], and, for comparative purposes, other cancer sites.

### Materials and methods

Data for the present report were abstracted from the Vaud Cancer Registry file, which includes incident cases of malignant neoplasms in the canton (Levi *et al*., 1992) whose population, according to the 1990 census, was about 600 000 inhabitants. The registry file is tumour based, and multiple primaries in the same person are entered separately. Most cases are registered repeatedly and from different institutions, thus improving completeness and accuracy of registration. The basic information available from the register comprises sociodemographic characteristics of the patient (i.e. age and sex), primary site and histological type of the tumour according to the standard *International Classification of Diseases for Oncology* (ICD-O; World Health Organization, 1976), and time of diagnostic confirmation (histological or clinical diagnosis). Passive and active follow-up is recorded, and each subsequent item of information concerning an already registered case is used to complete the record of that patient.

Since 1974, a registration scheme, applying the same standardised rules as for incident malignancies, has been implemented for carcinoma *in situ* and severe dysplasia of the uterine cervix (CIN III). All histological reports were scrutinised and reviewed when reporting a diagnosis of CIS.

After exclusion of all synchronous CIS and other cancers

**Table I** Age distribution of 2190 cases of histologically verified *in situ* carcinoma (CIS) of uterine cervix, corresponding incidence rates and person-years at risk by time since diagnosis (Vaud, Switzerland, 1974-93)

| | *Carcinoma* in situ, n | *Rate per 100 000* |
|---|---|---|
| Age group (years) | | |
| 0-19 | 16 | 1.0 |
| 20-29 | 642 | 77.7 |
| 30-39 | 859 | 101.0 |
| 40-49 | 412 | 54.8 |
| 50-59 | 135 | 21.9 |
| 60-69 | 87 | 16.0 |
| 70-79 | 30 | 7.1 |
| 80+ | 9 | 3.3 |
| Total, all ages | 2190 | 34.9[a] |
| Time since diagnosis (years) | | Person-years at risk by time since diagnosis |
| 1-4 | | 9319 |
| 5-9 | | 7035 |
| 10-14 | | 4438 |
| 15+ | | 1433 |
| Total | | 22 225 |

[a] Age-standardised rate on the world population.

Correspondence: F Levi
Received 9 April 1996; revised 13 May 1996; accepted 13 May 1996

Table II Observed (O) and expected (E) second primary cancers or groups of cancers following 2190 cases of *in situ* carcinoma of the uterine cervix according to time since diagnosis, and corresponding overall standardised incidence ratios (SIR) (Vaud, Switzerland, 1974–93)

| Site | | Years since diagnosis 1–4 | 5–9 | 10–14 | 15+ | Whole period | SIR (95% CI) |
|---|---|---|---|---|---|---|---|
| Mouth of pharynx | O | 1 | 1 | 4 | 5 | 1,1 | |
| oesophagus, lung, bladder | E | 1.5 | 1.6 | 1.4 | 0.6 | 5.1 | 2.2 (1.1–3.9) |
| Skin, non-melanoma | O | 5 | 10 | 9 | 5 | 29 | |
| | E | 5.1 | 5.3 | 4.5 | 1.9 | 16.9 | 1.7 (1.1–2.5) |
| Skin, melanoma | O | 1 | 1 | 0 | 1 | 3 | |
| | E | 1.1 | 1.0 | 0.8 | 0.3 | 3.2 | 0.9 (0.2–2.7) |
| Breast (females) | O | 3 | 6 | 7 | 2 | 18 | |
| | E | 7.3 | 7.8 | 6.5 | 2.6 | 24.4 | 0.8 (0.4–1.2) |
| Cervix uteri | O | 6 | 3 | 1 | 0 | 10 | |
| | E | 1.0 | 0.9 | 0.7 | 0.3 | 3.0 | 3.4 (1.6–6.3) |
| Corpus uteri | O | 3 | 0 | 1 | 1 | 5 | |
| | E | 1.0 | 1.1 | 1.0 | 0.5 | 3.5 | 1.4 (0.5–3.3) |
| Other sites | O | 6 | 8 | 3 | 2 | 19 | |
| | E | 7.0 | 7.2 | 6.2 | 2.7 | 23.1 | 0.8 (0.5–1.3) |
| Total, all sites | O | 25 | 29 | 25 | 16 | 95 | |
| | E | 23.8 | 24.8 | 20.7 | 8.7 | 77.9 | 1.2 (1.0–1.5) |

($n=8$, i.e. two cancers of the breast, four of the uterine corpus, one of the ovary and one of unspecified genital organs), the present series comprises a total of 2190 histologically (at least through a biopsy) confirmed CIS. The age range was 18–92 years (median age, 34 years). These cases of incident CIS were followed up to the end of 1993, for the occurrence of cancer, migration or death. Histological confirmation was performed in 100% of both the CIS and of the second primaries.

Calculation of expected numbers was based on site-, age- and calendar year-specific incidence rates, multiplied by the observed number of person–years at risk. The significance of the observed/expected ratios (standardised incidence ratio, SIR), and the corresponding 95% confidence interval (CI), was based on the exact Poisson distribution (Breslow and Day, 1987).

### Results

Table I gives the distribution of 2190 cases of CIS according to age, the corresponding incidence rate for the whole calendar period, and the person–years at risk in separate intervals of time since diagnosis, for a total of 22 225 person–years at risk.

Table II gives the observed and expected numbers of all neoplasms and of selected cancer sites in separate strata of years since diagnosis. Overall, 95 metachronous cancers were observed *vs* 77.9 expected, corresponding to a significant SIR of 1.2. The excess was larger 15 years or more after CIS diagnosis (16 observed, 8.7 expected, SIR = 1.8). Ten cases of invasive cervical cancer were observed *vs* 3.0 expected (SIR = 3.4), the excess incidence being largest in the first 5 years (six observed, one expected), and between 5 and 9 years (three observed, 0.9 expected) since CIS diagnosis, and levelling off thereafter.

Four major tobacco-related sites (lung, mouth or pharynx, oesophagus and urinary bladder) were grouped together. A total of 11 cases (one of the mouth or pharynx, one of the oesophagus, seven of the lung, and two of the bladder) were observed, *vs* 5.1 expected, corresponding to a significant SIR of 2.2. The excess was greater 10 years or longer after CIS diagnosis (four observed, 1.4 expected, SIR = 2.9, between 10 and 14 years; five observed, 0.6 expected, SIR = 8.3, $\geqslant 15$ years). There was also an excess of non-melanomatous skin cancers (29 observed, 16.9 expected, SIR = 1.7), the elevated risk being greatest 5 years after CIS diagnosis or longer, but not of skin melanoma (three observed *vs* 3.2 expected, SIR = 0.9). None of the other neoplasms considered, including breast (SIR = 0.8), corpus uteri (SIR = 1.4) or other miscellaneous sites (SIR = 0.8) showed excess incidence in women with CIS.

### Discussion

In this population, there was an excess of invasive cervical cancer following histologically confirmed CIS, which was however restricted to the 10 years after CIS diagnosis. This may partly be due to increased ascertainment but underlines the importance of adequate treatment and surveillance of women after CIN (La Vecchia *et al.*, 1995).

There was also a significant excess of tobacco-related neoplasms, which became evident 10 years after CIS diagnosis. This may be interpretable in terms of shared risk factors, since a role of tobacco in cervical carcinogenesis has long been suspected (Winkelstein, 1977; Brinton, 1992). In a review of 33 epidemiological studies of the relationship between cigarette smoking and cancer of the uterine cervix published over the last three decades (Winkelstein, 1990), 26 found a direct association. This association has also been reported for cervical intraepithelial neoplasia (including CIN III; Parazzini *et al.*, 1992, 1996; Szarewski *et al.*, 1996). Furthermore, nicotine and cotinine have been identified in cervical mucus, and their concentration was directly related to cigarette smoking (Hellberg *et al.*, 1988; Schiffman *et al.*, 1987; McCann *et al.*, 1992).

A significant excess of lung cancer was also observed following invasive cervical cancer in the same population (Levi *et al.*, 1993a), and rates from several tobacco-related neoplasms were also elevated in a large study of *in situ* cervical cancer from Norway (Bjorge *et al.*, 1995). Likewise, an increase for lung and bladder cancer incidence was observed in Denmark (Storm and Ewertz, 1985; Storm, 1988), and for lung, oral cavity, larynx and bladder in Connecticut following cervical cancer (Rabkin *et al.*, 1992).

It is also possible that human papilloma virus (HPV), the major identified risk factor for cervical neoplasms, is related to oral and pharyngeal, and, possibly, squamous cell carcinomas from other epithelia (IARC, 1995; Franceschi *et al.*, 1996; Maden *et al.*, 1992; Benamouzig *et al.*, 1992; Gissmann *et al.*, 1983).

The (non-melanomatous) skin cancer excess in the medium to long term following a CIS is more difficult to understand (although it has also been reported in other studies; Hartveit, 1988; Bjorge *et al.*, 1995). In this study, ascertainment bias is unlikely, since the excess risk became evident five or more years after CIS diagnosis. Again HPV (IARC, 1995), or subtle immunological impairment, may be aspects shared by cervical and skin carcinogenesis. A confounding effect of some lifestyle habits, chiefly recreational sun exposure, cannot be excluded.

Among the strengths of the study, there are its population basis, which should render any estimate relatively free from selection bias (Levi *et al.*, 1993*b*) and the complete histological confirmation of CIS cases. The absence of association with any of the other cancer sites is also reassuring with respect to surveillance bias, and provides relevant indications for the potential focus on a preventive and early diagnosis level for women diagnosed with CIS.

**Acknowledgements**
The contribution of the Swiss League against Cancer is gratefully acknowledged. We thank the Vaud Cancer Registry's staff.

### References

BENAMOUZIG R, PIGOT F, QUIROGA G, VALIDIRE P, CHAUSSADE S, CATALAN F AND COUTURIER D. (1992). Human papillomavirus infection in esophageal squamous-cell carcinoma in western countries. *Int. J. Cancer*, **50**, 549–552.

BJORGE T, HENNIG EM, SKARE GB, SOREIDE O AND THORESEN SO. (1995). Second primary cancers in patients with carcinoma *in situ* of the uterine cervix. The Norwegian experience 1970–1992. *Int. J. Cancer*, **62**, 29–33.

BRESLOW NE AND DAY NE. (1987). *Statistical Methods in Cancer Research*. Vol. II. *The Analysis of Cohort Studies*. IARC Scientific Publication No. 82. p. 71. IARC: Lyon.

BRINTON LA. (1992). Epidemiology of cervical cancer – overview. In *The Epidemiology of Cervical Cancer and Human Papillomavirus*, Munoz N, Bosch FX, Shah KV and Meheus A. (eds.), pp. 3–23. IARC: Lyon.

FRANCESCHI S, MUNOZ N, BOSCH F, SNIJDERS P AND WALBOOMERS MM. (1996). HPV and cancers of the upper aero-digestive tract. *Cancer Epidemiol. Biomarkers Prev.* (in press).

GISSMANN L, WOLNIK L, IKENBERG H, KOLDOVSKY U, SCHNURCH HG AND ZUR HAUSEN H. (1983). Human papillomavirus types 6 and 11 DNA sequences in genital and laryngeal papillomas and in some cervical cancers. *Proc. Natl Acad. Sci USA*, **80**, 560–563.

HARTVEIT F. (1988). Association of skin and other lesions with cervical dysplasia. *Oncology*, **45**, 103–106.

HELLBERG D, NILSSON S, HALEY NJ, HOFFMAN D AND WYNDER E. (1988). Smoking and cervical intraepithelial neoplasia: nicotine and cotinine in serum and cervical mucus in smokers and nonsmokers. *Am. J. Obstet. Gynecol.*, **158**, 910–913.

IARC. (1995). *IARC Monographs on the Evaluation of Carcinogenic Risks to Humans*, Vol. 64, *Human Papillomaviruses*, p. 409. IARC: Lyon.

LA VECCHIA C, LEVI F AND FRANCESCHI S. (1995). Screening for cancer, 1995: an update. *Ann. Oncol*, **6**, 537–541.

LEVI F, TE VC, RANDIMBISON L, LA VECCHIA C AND RANDRIAMIHARISOA A. (1992). Statistics from the Registry of the Canton of Vaud, Switzerland, 1983–1987. In *Cancer Incidence in Five Continents, Vol. VI*. Parkin DM, Muir CS, Whelan SL, Gao YT, Ferlay J and Powell J. (eds). IARC Scientific Publication No. 120. pp. 762–765. IARC: Lyon.

LEVI F, RANDIMBISON L, TE VC, ROLLAND-PORTAL I, FRANCESCHI S AND LA VECCHIA C. (1993*a*). Multiple primary cancers in the Vaud Cancer Registry, Switzerland, 1974–89. *Br. J. Cancer*, **67**, 391–395.

LEVI F, LA VECCHIA C, RANDIMBISON L AND FRANCESCHI S. (1993*b*). Incidence of infiltrating cancer following superficial bladder carcinoma. *Int. J. Cancer*, **55**, 419–421.

MCCANN MF, IRWIN DE, WALTON LA, HULKA BS, MORTON JL AND AXELRAD CM. (1992). Nicotine and cotinine in the cervical mucus in smokers, passive smokers and nonsmokers. *Cancer Epidemiol. Biomarkers Prev.*, **1**, 125–129.

MADEN C, BECKMANN AM, THOMAS DB, MCKNIGHT B, SHERMAN KJ, ASHLEY RL, COREY L AND DALING JR. (1992). Human papillomaviruses, herpes simplex viruses, and the risk of oral cancer in men. *Am. J. Epidemiol*, **135**, 1093–1102.

PARAZZINI F, LA VECCHIA C, NEGRI E, FEDELE L, FRANCESCHI S AND GALLOTTI L. (1992). Risk factors for cervical intraepithelial neoplasia. *Cancer*, **69**, 2276–2282.

PARAZZINI F, LA VECCHIA C, NEGRI E, DAL PINO D AND FEDELE L. (1996). Effect of smoking cessation on cervical lesion size. *Lancet*, **347**, 620.

RABKIN CS, BIGGAR RJ, MELBYE M AND CURTIS RE. (1992). Second primary cancers following anal and cervical carcinoma: evidence of shared etiologic factors. *Am. J. Epidemiol.*, **136**, 54–58.

SCHIFFMAN MH, HALEY NJ, FELTON JS, ANDREWS AW, KASLOW RA, LANCASTER WD, KURMAN RJ, BRINTON LA, LANNOM LB AND HOFFNANN D. (1987). Biochemical epidemiology of cervical neoplasia: measuring cigarette smoke constituents in the cervix. *Cancer Res.*, **47**, 3886–3888.

SCHMULEWITZ EY, WEISS NS AND SCHWARTZ SM. (1993). Cutaneous melanoma following cervical intra-epithelial neoplasia in western Washington State. *Cancer Causes Control*, **4**, 225–229.

STORM HH. (1988). Second primary cancer after treatment for cervical cancer. Late effects after radiotherapy. *Cancer*, **61**, 679–688.

STORM HH AND EWERTZ M. (1985). Second cancer following cancer of the female genital system in Denmark, 1943–80. *Natl Cancer Inst. Monogr.*, **68**, 331–340.

SZAREWSKI A, JARVIS MJ, SASIENI P, ANDERSON M, EDWARDS R, STEELE SJ, GUILLEBAUD J AND CUZICK J. (1996). Effect of smoking cessation on cervical lesion size. *Lancet*, **347**, 941–943.

WINKELSTEIN W JR. (1977). Smoking and cancer of the uterine cervix: hypothesis. *Am. J. Epidemiol.*, **106**, 257–259.

WINKELSTEIN. (1990). Smoking and cervical cancer–current status: A review. *Am. J. Epidemiol.*, **131**, 945–957.

WORLD HEALTH ORGANIZATION. (1976). *International Classification of Diseases for Oncology*; WHO: Geneva.